# Acoustic radiation force impulse elastography and liver fibrosis risk scores in severe obesity

**DOI:** 10.20945/2359-3997000000397

**Published:** 2021-11-11

**Authors:** Roberto Gomes da Silva, Maria Luiza Queiroz de Miranda, Paulo Eugênio de Araújo Caldeira Brant, Perla Oliveira Schulz, Maria de Fátima Araujo Nascimento, Joel Schmillevitch, Andrea Vieira, Wilson Rodrigues de Freitas, Luiz Arnaldo Szutan

**Affiliations:** 1 Faculdade de Ciências Médicas da Santa Casa de São Paulo Unidade de Gastroenterologia Departamento de Medicina São Paulo SP Brasil Departamento de Medicina, Unidade de Gastroenterologia, Faculdade de Ciências Médicas da Santa Casa de São Paulo, São Paulo, SP, Brasil; 2 Faculdade de Ciências Médicas da Santa Casa de São Paulo Departamento de Patologia São Paulo SP Brasil Departamento de Patologia, Faculdade de Ciências Médicas da Santa Casa de São Paulo, São Paulo, SP, Brasil; 3 Centro de Diagnóstico Schmillevitch São Paulo SP Brasil Centro de Diagnóstico Schmillevitch, São Paulo, SP, Brasil; 4 Faculdade de Ciências Médicas da Santa Casa de São Paulo Departamento de Cirurgia São Paulo SP Brasil Departamento de Cirurgia, Faculdade de Ciências Médicas da Santa Casa de São Paulo, São Paulo, SP, Brasil

**Keywords:** Non-alcoholic fatty liver disease, non-invasive markers, liver elastography, bariatric surgery

## Abstract

**Objective:**

Identifying significant fibrosis is crucial to evaluate the prognosis and therapeutic interventions in patients with nonalcoholic fatty liver disease (NAFLD). We assessed the performance of acoustic radiation force impulse (ARFI) elastography, APRI, FIB-4, Forns, NFS and BARD scores in determining liver fibrosis in severe obesity.

**Subjects and methods:**

A prospective study included 108 patients undergoing bariatric surgery. Liver biopsy specimens were obtained intraoperatively and classified according to the NAFLD Activity Score. Patients were assessed with serological markers and shear wave velocity of the liver was measured with the Siemens S2000 ultrasound system preoperatively. Optimal cut-off values were determined using the area under the receiver operating characteristic curves (AUROC).

**Results:**

In the entire cohort prevalence of NAFLD was 80.6%, steatohepatitis 25.9% and significant fibrosis 19.4%. The best tests for predicting significant fibrosis were FIB-4 and Forns scores (both AUROC 0.78), followed by APRI (AUROC 0.74), NFS (AUROC 0.68), BARD (AUROC 0.64) and ARFI (AUROC 0.62). ARFI elastography was successful in 73% of the patients. Higher body mass index (BMI) correlated with invalid ARFI measurements. In patients with BMI < 42 kg/m^2^, ARFI showed 92.3% sensitivity and 82,6% specificity for the presence of significant fibrosis, with AUROC 0.86 and cut-off 1.32 m/s.

**Conclusions:**

FIB-4 and Forns scores were the most accurate for the prediction of significant fibrosis in bariatric patients. Applicability and accuracy of ARFI was limited in individuals with severe obesity. In patients with BMI < 42 kg/m^2^, ARFI elastography was capable for predicting significant fibrosis with relevant accuracy.

## INTRODUCTION

Obesity prevalence is raising worldwide and it is significantly associated with an increased incidence of nonalcoholic fatty liver disease (NAFLD). This condition covers a histological spectrum that ranges from simple steatosis to nonalcoholic steatohepatitis (NASH), fibrosis and cirrhosis (1). Identifying the presence and severity of liver fibrosis in the NAFLD population is a priority. Liver biopsy (LB) pursues as the gold standard for diagnosing NAFLD and is the only safe means of staging fibrosis. However, it is costly, invasive, and carries a risk for complications that can be serious with a morbidity between 0.3 and 0.6% and mortality of 0.05%. In addition, it has been reported that inter- and intra-observer discrepancies occur at a rate of 10% to 20% (2). Consequently, several noninvasive methods have been proposed to stage liver fibrosis, including tests composed of routinely available measures and imaging techniques.

Such scores include the aspartate aminotransferase (AST) to platelet ratio index (APRI), the Fibrosis-4 (FIB-4) score, and the Forns index that have been not initially designed for NAFLD (3,4). Two simple scores that are specific to NAFLD-related fibrosis have also been developed; the NAFLD fibrosis score (NFS) and BARD test incorporate body mass index (BMI) and diabetes status into their formula (5,6). The performance of all these fibrosis risk scores have not been strongly validated in severe obesity. Among the imaging methods, transient elastography is currently the most used elastography technique in clinical practice and it exhibited good applicability in patients candidate to bariatric surgery (7). Acoustic radiation force impulse (ARFI) elastography is a new technique that rapidly measures tissue stiffness and it is necessary to question how it behaves in patients with morbid obesity (8).

In this study, the objective is to evaluate the performance of ARFI elastography, APRI, FIB-4, Forns, NFS and BARD scores to diagnose significant hepatic fibrosis in patients with severe obesity. We also attempt to identify whether modified cut-offs result in improved accuracy in morbid obesity.

## SUBJECTS AND METHODS

### Study population

We performed a single-centered and prospective study in 108 consecutive adult patients who were to undergo bariatric surgery at the Santa Casa Hospital Complex of São Paulo, Brazil, between July 2015 and August 2018. Patients were recruited in this research based on the following criteria: BMI > 35 kg/m^2^, documented failure of non-surgical weight loss programs, acceptable operative risk, supportive family and social environment, absence of substance abuse, and absence of uncontrolled psychotic or depressive disorder. The histologic criterion for the diagnosis of NAFLD was the presence of macrovesicular fatty changes in hepatocytes, with displacement of the nucleus to the edge of the cell. All patients were evaluated by a multidisciplinary committee made up of endocrinologists, psychiatrists, and surgeons.

Patients were excluded if they had any other liver diseases, including viral, medication-related, autoimmune, or familial/genetic, or had a history of excessive alcohol intake (women who had been drinking more than 20 g of alcohol per day and men who had been drinking more than 40 g of alcohol per day in the last year or more) and if there was insufficient liver tissue for the staging of fibrosis. This study was approved by the Hospital’s Internal Review Board and Research Ethics Committee (CAAE: 39401714.0.0000.5479) and is in accordance with the principles of the Declaration of Helsinki and its appendices. All participating individuals read and signed informed consent forms.

### Physical examination and serum biochemistry

Patients underwent a physical examination and medical history within 2 weeks of surgery. The weight and height of the patients were measured with a calibrated scale after the patients removed any items of clothing. Fasting blood tests were taken before surgery and analyzed in the same laboratory: AST, alanine aminotransferase (ALT), gamma glutamyl transpeptidase (GGT), platelet count, triglycerides, glucose, glycated hemoglobin, cholesterol, albumin, and international normalized ratio (INR). The upper limits of the normal (ULN) AST concentrations in patients was 33 IU/mL. Subjects with an elevated blood pressure (≥130/≥85 mmHg) documented on at least two clinic visits before surgery were considered as hypertensive or any patient taking antihypertensive agents. Criteria for type 2 diabetes were based on the American Diabetes Association (9): fasting blood glucose >126 mg/dL or 2h glucose tolerance test >200 mg/dL. Fasting blood glucose of >100 mg/dL was considered to be hyperglycemic. Based on these results, we calculated the following tests for predicting liver fibrosis:

- APRI score = [(AST/ULN) x 100] / platelet count 10^9^/L (10);
- Forns score = 7.811 - 3.131 x ln [platelet count (10^9^/L)] + 0.781 x ln[(GGT (IU/L)] + 3.467 x ln [age(years)] – 0.014 [cholesterol (mg/dL)] (11);
- FIB-4 score = [age (years) x AST (IU/L)] / [platelet count (10^9^/L) x ALT (IU/L)^1/2^] (12);
- NFS score = −1.675 + 0.037 × age [years] + 0.094 ×BMI + 1.13 × diabetes status + 0.99 × AST/ALT – 0.013 × platelet [109/L] – 0.66 × albumin [g/dL] (5);
- BARD score = BMI ≥ 28 = 1 point; AST/ALT ratio ≥0.8 = 2 points; Diabetes status = 1 point (6).

### Histological assessment

Liver biopsies were performed intraoperatively using a Tru-cut needle. All tissues were evaluated by an experienced pathologist who was unaware of the clinical data. A diagnosis of NAFLD was made after a thorough histologic evaluation of each patient’s LB sample as well as looking carefully for evidence of other liver disorders, such as autoimmune hepatitis, hepatitis C, hepatitis B, iron overload, and primary biliary cirrhosis. Fibrosis was staged acceding to the NASH Clinical Research Network scoring system (13) in this manner: stage 0 (F0), absence of fibrosis; stage 1 (F1), perisinusoidal or portal; stage 2 (F2), perisinusoidal and portal/periportal; stage 3 (F3), septal or bridging fibrosis; stage 4 (F4), cirrhosis. The grade of steatosis was also defined according to Kleiner and cols. (13): S0, <5%; S1, 5%-33% (mild); S2, 34%-66% (moderate); and S3, >67% (marked). Disease activity was quantified using the NAFLD activity score (NAS), which is based on the severity of steatosis (0-3), inflammation (0-3), and hepatocellular ballooning (0-2).

### Ultrasound elastography

ARFI imaging was performed at least 4 weeks before the scheduled bariatric surgery in all patients using an ACUSON S2000™ Ultrasound System (Siemens Medical Solutions, Inc.) equipped with a 4-1 MHz multi-frequency convex probe. All procedures were conducted by the same radiologist that had significant experience in digestive system ultrasonography and 5 years’ experience in elastography and who was blinded to the clinical, serological and histological data. ARFI elastography was executed in such a way: the right lobe of the liver was appraised through an intercostal space while the patient laid in the dorsal decubitus position with their right arm in maximum abduction. The operator located the probe up the segment eight of the right lobe, distant from motion and hepatic vessels, about 2 cm from the liver capsule, at a depth between 4.0 and 6.0 cm. Ten shear wave speed (SWS) acquisitions were acquired for each subject, with one acquisition per breath-hold. For each acquisition, if the proscribed measurement quality threshold was not achieved by the ultrasound scanner, it showed an error message. In this circumstance, no SWS value was displayed, and the acquisition was considered invalid. Exams with less than 6 valid measurements or an interquartile range (IQR) >30% of the median liver stiffness measurement value were considered to have failed ARFI and were excluded from added analysis.

### Data analysis

Statistical study was performed using MedCalc software version 19.2.1 for Windows (Ostend, Belgium). Continuous variables were expressed as mean ± standard deviation (SD). Student t test was used for parametric data and Mann-Whitney U test for nonparametric data. Categorical variables were expressed as numbers (with percentages) and Pearson’s chi-squared tests were used. For correlation analysis, Spearman’s rank correlation coefficient was calculated. The receiver operating characteristic curve (AUROC) was measured, as well as calculates of diagnostic accuracy: sensitivity (Se), specificity (Sp), positive predictive value (PPV) and negative predictive value (NPV). Optimal cutoff values for differentiating F2-4 diseases were calculated by finding the highest Youden Index. All tests were performed with a significance level of p < 0.05. The statistical review of the study was performed by a biomedical statistician.

## RESULTS

### Study population

In all, 123 consecutive patients were screened to undergo bariatric surgery. Eight subjects were excluded on the basis of having other causes of liver disease or being on medications with potential for causing liver damage. Four patients were excluded due to an inadequate LB for a histological diagnosis and staging. Three patients denied consent for the research. A total of 108 subjects were included in the study. Comparative data of 108 patients with severe obesity studied are summarized in Table 1. Nine (8.3%) patients with Class II obesity (BMI 35-39.9), 67 (62.1%) Class III obesity (BMI 40-49.9), 27 (25%) superobese (BMI 50-59.9), and 5 (4.6%) super superobese (BMI ≥ 60). AST value had been normal in 87 (80.6%) patients and ALT value had been normal in 73 (67.6%) patients. The histological findings are provided in Table 2. Prevalence of NAFLD was 80.6% in the entire cohort. Some degree of fibrosis (F1-4) was identified in 46 (42.6%) participants, 21 (19.4%) had significant fibrosis (F2-4). The presence of advanced fibrosis (F3-4) was found in 9 (8.3%) patients, 28 (25.9%) showed an NAS ≥ 5, which was considered to be indicative of a NASH diagnosis, while 25 patients (23.1%) had a borderline NAS of 3-4.

**Table 1:** Baseline demographics, measurements and comparison between those with no/minimal fibrosis (F0-1) and significant fibrosis (F2-4)

	Fibrosis staging			
	Patient population	F0-F1	F2-F4	P
n	108	87 (81%)	21 (19%)	
Age (years)	44.6 ± 10.4	43.3	50	0.008
Gender female	86 (79%)	70 (80%)	16 (76%)	0.639
BMI (kg/m^2^)	46.9 ± 6.6	47.2	45.6	0.342
Platelet (10^9^/L)	261.1 ± 76.1	272.9	212.1	0.001
ALT/ULN	0.9 ± 0.6	0.9	1.1	0.105
AST/ULN	0.8 ± 0.6	0.7	1.2	0.001
GGT (U/L)	49.9 ± 28.2	45.4	66.5	0.078
Albumin (g/mL)	4.3 ± 0.4	4.2	4.4	0.056
Cholesterol (mg/dL)	191.1 ± 39.7	193.7	180.6	0.177
Type II diabetes	39 (36%)	27 (31%)	12 (57%)	0.025
Hyperglycemia	55 (51%)	42 (48%)	13 (61%)	0.084
Hypertension	69 (64%)	57 (66%)	12 (57%)	0.524
Hypercholesterolemia	63 (58%)	50 (57%)	13 (61%)	0.889
Hypertriglyceridemia	45 (42%)	37 (43%)	8 (38%)	0.912
Low HDL	80 (74%)	65 (75%)	15 (71%)	0.703
APRI	0.39 ± 0.6	0.30	0.77	0.001
FIB-4	1.02 ± 1.2	0.79	1.99	<0.0001
Forns	3.77 ± 1.8	3.41	5.26	<0.0001
NFS	- 0.25 ± 1.4	- 0.45	0.55	0.004
BARD ≥ 2	73 (68%)	56 (64%)	17 (81%)	0.049
ARFI	1.47 ± 0.7	1.43	1.80	0.061

Values expressed in mean ± standard deviation or numbers (percentages). AST: aspartate aminotransferase; ALT: alanine aminotransferase; BMI: Body mass index; GGT: gamma-glutamyl-transferase; ULN: upper limit of normal.

**Table 2:** Histological features of the 108 individuals subjected to bariatric surgery according to Scoring System “NASH Clinical Research Network”

		Number of patients (%)
Steatosis grade:		
	S1 (5%-33%)	43 (39.8%)
	S2 (34%-66%)	16 (14.8%)
	S3 (>66%)	28 (25.9%)
NAFLD activity score (NAS):		
	NAS 1-2 points	34 (31.5%)
	NAS 3-4 points	25 (23.1%)
	NAS ≥ 5 points	28 (25.9%)
Histologic fibrosis stage:		
	F0	62 (57.5%)
	F1	25 (23.1%)
	F2	12 (11.1%)
	F3	4 (3.7%)
	F4	5 (4.6%)

### Predicting significant fibrosis

To correlate the noninvasive markers of fibrosis with the results of the liver biopsies, we isolated the patients into two groups: no/minimal fibrosis (F0-F1, n = 87, 80.6%) and significant fibrosis (F2-F4, n = 21, 19.4%). The variables that differed significantly between these two groups were age, platelet count, AST level and the presence of type 2 diabetes mellitus (Table 1). The ALT and GGT levels, gender and BMI did not differ significantly between the groups.

### Performance of ARFI elastography in patients with severe obesity

**Figure 1:**
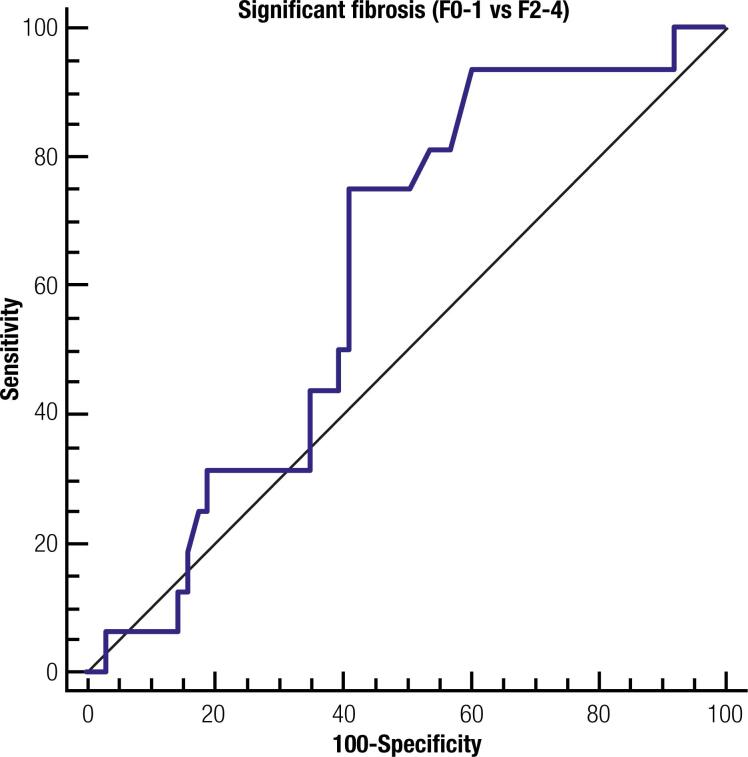
Receiver operator characteristic curve for ARFI elastography for the diagnosis of significant fibrosis (AUROC 0.62 ± 0.07, IC 95% 0.51-0.73, p 0.08).

The mean SWV by ARFI was 1.47 m/s (median = 1.31m/s). Receiver operating characteristic analysis showed no significant area under the curve (AUROC = 0.62 [95% CI: 0.51 - 0.73], p = 0.08) for diagnosis of significant fibrosis using SWV (Figure 1). The presence of significant fibrosis was diagnosed by a cut-off value of 1.32 m/s, ARFI measurements had 75% Se, 58.7% Sp, 31.6% PPV and 90.2% NPV. Valid SWV could be acquired in all subjects; however, ARFI failure was observed in 29 patients. Twenty of these patients had less than 6 valid measurements. In another 9 patients, liver stiffness analyses were not reliable (IQR/median > 0.30). Thus, ARFI elastography was successful in 73.1% of the patients. The interval between ARFI imaging and LB was 14.3 ± 8.4 days.

**Figure 2:**
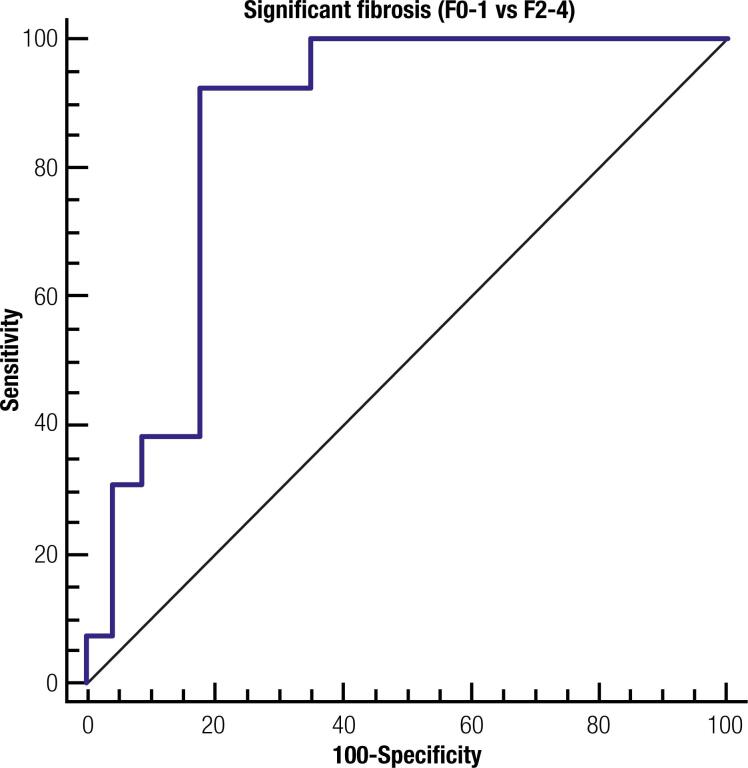
Receiver operator characteristic curve for ARFI elastography for the diagnosis of significant fibrosis in patients with body mass index < 42 kg/m^2^ (AUROC 0.86 ± 0.06, IC 95% 0.71-0.95, p < 0.0001).

ARFI failure was correlated with widening BMI, with 10% for BMI < 42 kg/m^2^ and 36.8% for BMI ≥ 42 kg/m^2^ (p < 0.05). In patients with no/minimal fibrosis, higher median ARFI values were detected in individuals with BMI ≥ 42 kg/m^2^. On the other hand, the median ARFI sonoelastography velocity did not reveal a stepwise change in the necroinflammatory activity. Additionally, no significant correlation was found among the percentage of steatosis obtained in the histopathological study and the ARFI measurements. We completed an analysis of the impact of BMI on the applicability and accuracy of ARFI elastography. In patients with BMI < 42 kg/m^2^ (40 subjects), ARFI measurements showed 92.3% Se and 82,6% Sp for the presence of significant fibrosis, with AUROC 0.86 [95% CI: 0.71 - 0.95], using the same cut-off value of 1.32 m/s (Figure 2).

### Accuracy of non-invasive composite scores: a new cut-offs proposal

**Figure 3:**
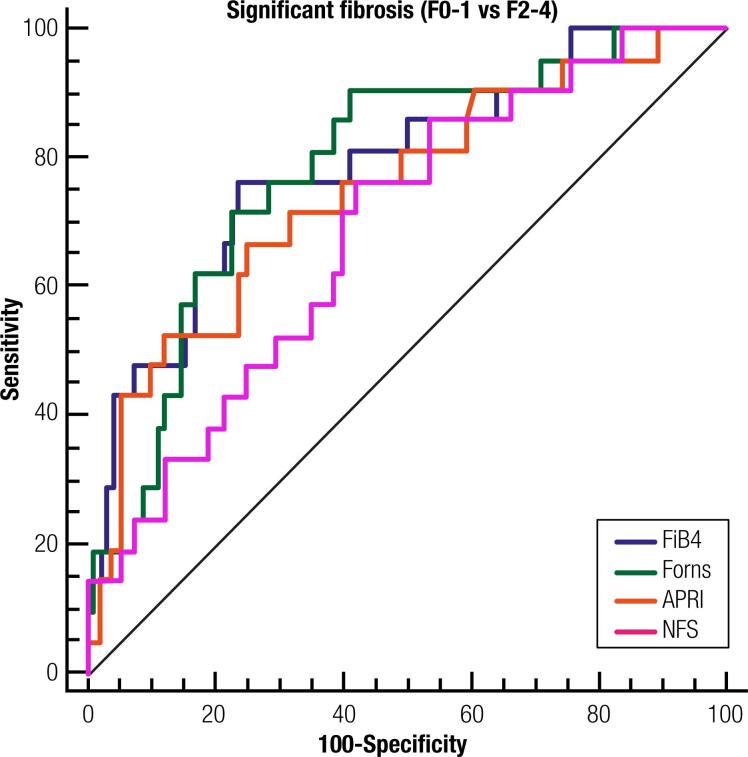
Receiver operator characteristic curves for presence of significant fibrosis using risk scores. (FIB4: AUROC 0.78 ± 0.06, IC 95% 0.69-0.86, p < 0.0001; Forns: AUROC 0.78 ± 0.05, IC 95% 0.69-0.85, p < 0.0001; APRI: AUROC 0.74 ± 0.06, IC 95% 0.65-0.82, p 0.001; NFS: AUROC 0.68 ± 0.06, IC 95% 0.59-0.77, p 0.004).

All scores were significantly correlated with the severity of fibrosis. The Forns score showed the strongest correlation (r = 0.392). The AUROC for the Forns and FIB-4 scores had a moderate ability for differentiating significant fibrosis, both with AUROC 0.78, p < 0.001 (Figure 3). The remaining scores had lower AUROC values: APRI (AUROC 0.74), NFS (AUROC 0.68) and BARD score (AUROC 0.64).

Based on the standard threshold values, sensitivities were poor for Forns, FIB-4, APRI and NFS scores. When thresholds were modified to optimize detection of significant fibrosis, the cut-offs were significantly lower than those in the literature (Table 3). The Forns index cut-off decreased from 6.9 to 3.44 and FIB-4 from 3.25 to 0.85. Subsequently, a greater proportion of significant fibrosis was identified, which resulted in a substantially improved sensitivity. The negative predictive values were also improved for each test. BARD score showed 80.9% Se, 35.6% Sp, 23.3% PPV and 88.6% NPV for the presence of significant fibrosis using the cut-off value ≥ 2 points.

**Table 3:** New cut-offs proposed for presence of significant fibrosis in subjects with severe obesity, compared with those described in literature (n = 108)

		AUROC	Se (%)	Sp (%)	PPV (%)	NPV (%)
APRI						
	Literature cut-off: 0.70		28.6	94.3	54.5	84.5
	Cut-off proposed: 0.27	0.74	66.7	74.7	38.9	90.3
FIB-4						
	Literature cut-off: 3.25		14.3	97.7	60	82.5
	Cut-off proposed: 0.85	0.78	76.2	75.9	43.2	93
Forns						
	Literature cut-off: 6.90		19.1	97.7	66.7	83.3
	Cut-off proposed: 3.44	0.78	90.5	58.6	34.5	96.2
NFS						
	Literature cut-off: 0.67		42.9	77	31	84.8
	Cut-off proposed: 0.37	0.68	52.4	70.1	29.7	85.9

Se: sensitivity; Sp: specificity; PPV: positive predictive value; NPV: negative predictive value. AUROC: area under the receiver operating characteristic curve.

## DISCUSSION

Over the last two decades, the well-known limitations and risks of LB have stimulated the search for non-invasive approaches to assess the degree of fibrosis in patients with liver disease. Although non-invasive tests are progressively applied in the appraisal of liver fibrosis, clinicians must elect between diversified methods (14). This study unprecedentedly evaluated the performance of five fibrosis risk scores and ARFI elastography in 108 individuals with severe obesity that were scheduled for bariatric surgery. The preoperative characterization of hepatic fibrosis is of great importance in this population because it contributes to peri-intervention risk assessment and may influence the strategy of treatment (15).

We confirmed a high prevalence of NAFLD (80.6%), being in agreement with the literature (16). In addition, there was a higher proportion of diabetic patients in the significant fibrosis group, a finding that has also been reported in other populations (17). Another important finding was the unsatisfactory performance of Forns, FIB-4, APRI and NFS scores using standard thresholds. A simple modification of cut-off values substantially improved their accuracy for differentiating F2-4 fibrosis in our population of individuals with severe obesity. Forns and FIB-4 scores obtained the best accuracy for the prediction of significant fibrosis in this population, including a high sensitivity and NPV up to 93%.

The Forns index was initially used in hepatitis C patients, for whom the predicted accuracy for significant fibrosis was reported to be between 50% and 83% using a cut-off value of 6.9 (11). Recently, Ooi and cols. reported in a study of NAFLD patients with obesity, a PPV and NPV for significant fibrosis of 44% and 93%, respectively and the optimal cut-off point value (3.50) was similar as our sample (18). The FIB-4 system was initially used in human immunodeficiency virus and hepatitis C virus co-infected patients and was subsequently validated for NALFD. It has shown interesting results in studies published from around the word (19). For values higher than 3.25 in a comparison of fibrosis markers in 541 NAFLD patients, FIB-4 acquired the highest AUROC of 0.80, with NPV and PPV of 90% and 80%, respectively, in predicting advanced fibrosis (20). Pérez-Gutiérrez and cols., using the same cut-off value for predictions of severe fibrosis, in a Latin population, obtained lower PPV of 26% and 53% Se (21). Recently, a retrospective study with 323 individuals with obesity undergoing bariatric surgery obtained a diagnostic accuracy of 0.90 to predict advanced fibrosis (22). In the current study, using a similar cut-off value (0.85 vs 0.74), FIB-4 was more specific (76% vs 52%) and less sensitive (76% vs 86%), compared to the data reported by Ooi and cols. in morbid obesity (18).

The APRI ratio is not expensive and is accessible to all clinicians. Using this score, Cales and cols. demonstrated an AUROC of 0.87 for significant fibrosis in a study of 235 NAFLD patients (23). Recent Brazilian studies have shown that it was able to exclude clinically significant fibrosis with 85.9% in patients submitted to bariatric surgery and it was the best predictor of advanced liver disease in severe obesity (24,25). Similar levels of accuracy for the prediction of significant fibrosis have also been reported for APRI and BARD in 242 NAFLD subjects from Australia. The AUROC values contrasted to our cohort for APRI were 0.71 versus 0.74, respectively, and 0.61 versus 0.64 for BARD (26). The BARD test combines the BMI, AST⁄ALT ratio and the presence of diabetes variables into a weighted sum to generate a score between 0 and 4. In the original study, a score of 2-4 was correlated with an odds’ ratio for advanced fibrosis of 17 (6). Lower accuracies have been obtained in subsequent studies. Ruffillo and cols. reported an AUROC of 0.67 for the diagnosis of advanced fibrosis (27). Similar to our results, Ooi and cols. indicated an AUROC of 0.58 for the diagnosis of significant fibrosis in patients with severe obesity (18). The present data show that the BARD score has poor diagnostic value for F2-4 fibrosis.

Scores that included BMI were less diagnostic of fibrosis in this cohort. Given this bariatric population, all had a high BMI; this measure is unlikely to contribute significantly in differentiating the presence of fibrosis. Consequently, NFS and BARD scores have showed poor diagnostic power in the current study. The NFS is composed of six variables that was formulated using a panel of 733 NAFLD subjects across diverse international centers. Cales and cols. reported an AUROC of 0.88 for predicting the presence of significant fibrosis (23). The score also exhibited ample accuracy for excluding significant fibrosis in subjects with morbid obesity and NPV in the range of 85%-88% (18,28). The values attained in our study are comparable with those obtained in patients undergoing bariatric surgery.

In the current study, we also observed ample variation in the ARFI values. This variability resulted in an overestimation of liver fibrosis in most individuals with obesity. In contrast to many non-bariatric cohorts, in which ARFI achieved high diagnostic accuracies for the detection of liver fibrosis in NAFLD (29,30), ARFI did not contribute any diagnostic benefit in patients with BMI ≥ 42m^2^/kg. Discordant values may be related to higher BMI and increasing hepatic steatosis. We hypothesize that our findings may occur because in ARFI elastography tissue displacement cannot be efficiently induced secondary to dampening of acoustic push pulse through the dense subcutaneous fat layers of patients with morbid obesity. Latter studies have also indicated that obesity may negatively influences the efficiency of ARFI for diagnosing fibrosis in NAFLD patients (31,32). Cui and cols. reported that the AUROC of ARFI declined further to 0.53 in the cohort of individuals with morbid obesity (33). Palmeri and cols. revealed that the rate of successful liver stiffness measurement was 100% in patients with BMI measurements of less than 23 kg/m^2^, 91% in those with BMI measurements of 23 to less than 30 kg/m^2^, 80% in those with BMI measurements of 30-40 kg/m^2^, and 58% in those with BMI measurements of more than 40 kg/m^2^(34).

Our data are contrary to a study from Guzman-Aroca and cols. They did not report problems in performing ARFI in morbid obesity (35). In contrast, the research recruited patients with lower BMI (mean 44.3 kg/m^2^) and a higher prevalence of NASH (33%) than we recognized in our cohort. On the other hand, in our patients with BMI < 42 kg/m^2^, the AUROC of ARFI elastography for significant fibrosis prediction was comparable to that of other prospective studies, 0.86 vs 0.89 – 0.94 (29,35). For a cut-off value of 1.32 m/s, the technique had 92% Se and 83% Sp. Further prospective studies on larger groups of patients are required to establish its role in this setting.

This study has limitations. First, our patients had lower rates of advanced fibrosis. A possible explanation may be our recruitment of consecutive patients with obesity that are considered high risk but have not been preselected based on a known diagnosis of NAFLD. Second, it was performed at a center that is highly specialized for both clinical and radiological research, and the generalizability of its results to other settings requires validation in a multicenter setting. In addition, the diagnostic accuracy of ARFI is reported to be operator-dependent and may be subject to interoperator and intraoperator variability, although in our study all ARFI was performed by a single experienced investigator.

In conclusion, we found that Forns and FIB-4 were the tests with the best performance for the diagnosis of significant liver fibrosis in patients with severe obesity. Furthermore, we propose new cut-offs of the most common noninvasive indexes to detect significant fibrosis in subjects with morbid obesity. Finally, we have shown that higher BMI negatively affected the applicability of ARFI for diagnosing fibrosis in NAFLD patients. In patients with BMI < 42 kg/m^2^, ARFI elastography was capable for predicting significant fibrosis with relevant accuracy.

## References

[1] Chang Y, Jung HS, Cho J, Zhang Y, Yun KE, Lazo M (2016). Metabolically Healthy Obesity and the Development of Nonalcoholic Fatty Liver Disease. Am J Gastroenterol.

[2] Bedossa P (2018). Diagnosis of non-alcoholic fatty liver disease/non-alcoholic steatohepatitis: Why liver biopsy is essential. Liver Int.

[3] Subasi CF, Aykut UE, Yilmaz Y (2015). Comparison of noninvasive scores for the detection of advanced fibrosis in patients with nonalcoholic fatty liver disease. Eur J Gastroenterol Hepatol.

[4] Bremer R, Pontes IC, Alves PML (2017). Can FIB4 and NAFLD fibrosis scores help endocrinologists refer patients with non-alcoholic fat liver disease to a hepatologist?. Arch Endocrinol Metab.

[5] Angulo P, Hui JM, Marchesini G, George J, Farrell GC, Enders F (2007). The NAFLD fibrosis score: a noninvasive system that identifies liver fibrosis in patients with NAFLD. Hepatology.

[6] Harrison SA, Oliver D, Arnold HL, Gogia S, Neuschwander-Tetri BA (2008). Development and validation of a simple NAFLD clinical scoring system for identifying patients without advanced disease. Gut.

[7] Takahashi H, Ono N, Eguchi Y, Eguchi T, Kitajima Y, Kawaguchi Y (2010). Evaluation of acoustic radiation force impulse elastography for fibrosis staging of chronic liver disease: a pilot study. Liver Int.

[8] Wan T, Köhn N, Kröll D, Berzigotti A (2021). Applicability and Results of Liver Stiffness Measurement and Controlled Attenuation Parameter Using XL Probe for Metabolic-Associated Fatty Liver Disease in Candidates to Bariatric Surgery. A Single-Center Observational Study. Obes Surg.

[9] American Diabetes Association (2012). Diagnosis and classification of diabetes mellitus. Diabetes Care.

[10] Wai CT, Greenson JK, Fontana RJ, Kalbfleisch JD, Marrero JA, Conjeevaram HS (2003). A simple noninvasive index can predict both significant fibrosis and cirrhosis in patients with chronic hepatitis C. Hepatology.

[11] Forns X, Ampurdanes S, Llovet JM, Aponte J, Quinto L, Martinez-Bauer E (2002). Identification of chronic hepatitis C patients without hepatic fibrosis by a simple predictive model. Hepatology.

[12] Vallet-Pichard A, Mallet V, Nalpas B, Verkarre V, Nalpas A, Dhalluin-Venier V (2007). FIB-4: an inexpensive and accurate marker of fibrosis in HCV infection. Comparison with liver biopsy and fibrotest. Hepatology.

[13] Kleiner DE, Brunt EM, Van Natta M, Behling C, Contos MJ, Cummings OW (2005). Design and validation of a histological scoring system for non-alcoholic fatty liver disease. Hepatology.

[14] Chalasani N, Younossi Z, Lavine JE, Charlton M, Cusi K, Rinella M (2018). The diagnosis and management of nonalcoholic fatty liver disease: Practice guidance from the American Association for the Study of Liver Diseases. Hepatology.

[15] Lassailly G, Caïazzo R, Pattou F, Mathurin P (2013). Bariatric surgery for curing NASH in the morbidly obese?. J Hepatol.

[16] Coccia F, Testa M, Guarisco G, Bonci E, Di Cristofano C, Silecchia G (2020). Noninvasive assessment of hepatic steatosis and fibrosis in patients with severe obesity. Endocrine.

[17] Hashiba M, Ono M, Hyogo H, Ikeda Y, Masuda K, Yoshioka R (2014). Glycemic variability is an independent predictive factor for development of hepatic fibrosis in nonalcoholic fatty liver disease. PLoS One.

[18] Ooi GJ, Burton PR, Doyle L, Wentworth JM, Bhathal PS, Sikaris K (2017). Modified thresholds for fibrosis risk scores in nonalcoholic fatty liver disease are necessary in the obese. Obes Surg.

[19] Dowman JK, Tomlinson JW, Newsome PN (2011). Systematic review: the diagnosis and staging of non-alcoholic fatty liver disease and non-alcoholic steatohepatitis. Aliment Pharmacol Ther.

[20] Shah AG, Lydecker A, Murray K, Tetri BN, Contos MJ, Sanyal AJ (2009). Comparison of noninvasive markers of fibrosis in patients with nonalcoholic fatty liver disease. Clin Gastroenterol Hepatol.

[21] Pérez-Gutiérrez OZ, Hernández-Rocha C, Candia-Balboa RA, Arrese MA, Benítez C, Brizuela-Alcántara DC (2013). Validation study of systems for noninvasive diagnosis of fibrosis in nonalcoholic fatty liver disease in Latin population. Ann Hepatol.

[22] de Carli MAL, de Carli LA, Correa MB, Junqueira G, Tovo CV, Coral GP (2020). Performance of noninvasive scores for the diagnosis of advanced liver fibrosis in morbidly obese with nonalcoholic fatty liver disease. Eur J Gastroenterol Hepatol.

[23] Cales P, Laine F, Boursier J, Deugnier Y, Moal V, Oberti F (2009). Comparison of blood tests for liver fibrosis specific or not to NAFLD. J Hepatol.

[24] Didoné CN, Reginatto CJ, Ivantes CAP, Strobel R, Percicote AP, Petenusso M (2021). Comparison between non-invasive methods and liver histology to stratify liver fibrosis in obese patients submitted to bariatric surgery. Obes Res Clin Pract.

[25] de Cleva R, Duarte LF, Crenitte MRF, de Oliveira CPM, Pajecki D, Santo MA (2016). Use of noninvasive markers to predict advanced fibrosis/cirrhosis in severe obesity. Surg Obes Relat Dis.

[26] Adams LA, George J, Bugianesi E, Rossi E, De Boer WB, van der Poorten D (2011). Complex non-invasive fibrosis models are more accurate than simple models in non-alcoholic fatty liver disease. J Gastroenterol Hepatol.

[27] Ruffillo G, Fassio E, Alvarez E, Landeira G, Longo C, Domínguez N (2011). Comparison of NAFLD fibrosis score and BARD score in predicting fibrosis in nonalcoholic fatty liver disease. J Hepatol.

[28] Qureshi K, Clements RH, Abrams GA (2008). The utility of the “NAFLD fibrosis score” in morbidly obese subjects with NAFLD. Obes Surg.

[29] Fierbinteanu Braticevici C, Sporea I, Panaitescu E, Tribus L (2013). Value of acoustic radiation force impulse imaging elastography for non-invasive evaluation of patients with nonalcoholic fatty liver disease. Ultrasound Med Biol.

[30] Friedrich-Rust M, Romen D, Vermehren J, Kriener S, Sadet D, Herrmann E (2012). Acoustic radiation force impulse-imaging and transient elastography for non-invasive assessment of liver fibrosis and steatosis in NAFLD. Eur J Radiol.

[31] Praveenraj P, Gomes RM, Basuraju S, Kumar S, Senthilnathan P, Parathasarathi R (2016). Preliminary Evaluation of Acoustic Radiation Force Impulse Shear Wave Imaging to Detect Hepatic Fibrosis in Morbidly Obese Patients Before Bariatric Surgery. J Laparoendosc Adv Surg Tech A.

[32] Karlas T, Dietrich A, Peter V, Wittekind C, Lichtinghagen R, Garnov N (2015). Evaluation of Transient Elastography, Acoustic Radiation Force Impulse Imaging (ARFI), and Enhanced Liver Function (ELF) Score for Detection of Fibrosis in Morbidly Obese Patients. PLoS One.

[33] Cui J, Heba E, Hernandez C, Haufe W, Hooker J, Andre MP (2016). Magnetic resonance elastography is superior to acoustic radiation force impulse for the diagnosis of fibrosis in patients with biopsy-proven nonalcoholic fatty liver disease: A prospective study. Hepatology.

[34] Palmeri ML, Wang MH, Rouze NC, Abdelmalek MF, Guy CD, Moser B (2011). Noninvasive evaluation of hepatic fibrosis using acoustic radiation force-based shear stiffness in patients with nonalcoholic fatty liver disease. J Hepatol.

[35] Guzman-Aroca F, Frutos-Bernal MD, Bas A, Luján-Mompeán JA, Reus M, Berná-Serna JD (2012). Detection of non-alcoholic steatohepatitis in patients with morbid obesity before bariatric surgery: preliminary evaluation with acoustic radiation force impulse imaging. Eur Radiol.

